# Unveiling consumer interest and regional disparities: comparative analysis of online search trends for penile aesthetic procedures

**DOI:** 10.1093/sexmed/qfaf013

**Published:** 2025-03-08

**Authors:** Elia Abou Chawareb, Jasmin Banton, Muhammed A M Hammad, Supanut Lumbiganon, Babak Azad, Jake Miller, Faysal A Yafi

**Affiliations:** Department of Urology, University of California, Irvine, CA 92617, United States; New York Institute of Technology College of Osteopathic Medicine, Jonesboro, AR 72401, United States; Department of Urology, University of California, Irvine, CA 92617, United States; Department of Urology, University of California, Irvine, CA 92617, United States; Department of Surgery, Faculty of Medicine, Khon Kaen University, Khon Kaen 40002, Thailand; Department of Urology, University of California, Irvine, CA 92617, United States; Department of Urology, University of California, Irvine, CA 92617, United States; Department of Urology, University of California, Irvine, CA 92617, United States

**Keywords:** minimally invasive surgical procedures, penis, plastic surgery, sexual health, trends

## Abstract

**Background:**

Aesthetic procedures for penile enhancement, such as the Penuma silicone sleeve implant, have gained increasing attention for addressing concerns like perceived size, buried or retractile penis, and mild curvature.

**Aim:**

To assess the online search interest over time for penile enhancement modalities, analyze the trends, and explore any regional disparities in search patterns.

**Methods:**

Google Trends data from June 18, 2018, to June 11, 2023, were utilized to analyze search interest for Penuma, penile implant, penile girth, Hyaluronic acid (HLA) injection, and penis injection. The results were compared to the trend data for Penuma from 2004 to 2023. Trendlines were generated to assess the changes in search interest over time and determine if they followed a random or polynomial trend. The highest search interest locations were identified for each term, and the corresponding regional gross domestic product values were collected.

**Outcomes:**

Search interest was assessed in terms of volume, temporal trends, and regional disparities.

**Clinical Implications:**

Understanding regional and temporal search patterns for penile enhancement can guide healthcare professionals and policymakers in developing targeted educational initiatives and allocating resources to meet patient needs.

**Strengths and Limitations:**

The use of Google Trends provides a comprehensive and real-time assessment of public interest over a broad timeframe and geographic range. However, search interest data may not fully capture actual patient behaviors or clinical demand, and the analysis relies on assumptions about search terms accurately reflecting consumer intent.

**Results:**

Penuma initially garnered interest upon its introduction in 2004 but experienced a decline until around March 2021. Comparing Penuma with other terms, general searches for penile implant and penile girth exhibited significantly higher interest than Penuma. The trendlines indicated increasing search interest for penile implant and HLA injection, while Penuma demonstrated a declining trend. In terms of regional disparities, the highest search interest for Penuma was observed in San Antonio, TX, while penile girth searches were highest in Oklahoma City, OK. Penile implant searches were prominent in Mobile, AL, and Birmingham, AL. Notably, HLA injection searches peaked in New York, NY, and penis injection searches were most prevalent in Los Angeles, CA.

**Conclusion:**

This study reveals that online interest in Penuma lags other penile enhancement terms, with notable regional disparities in search patterns. These findings underscore the need for further research to understand the factors influencing these trends and to help healthcare professionals tailor educational efforts and resources to diverse consumer needs.

## Introduction

As urologic concerns gain more attention, many people turn to the internet for information regarding procedures or conditions that may be seen as “taboo.” Many men have insecurities regarding their penises, and this can result in not only low self-esteem but also prevent them from enjoying sexual activities.^1^ A large study involving 25 592 men revealed that 45% of participants desired a larger penis size.^2^ Because of this, there is an increased interest in medical interventions to increase penile girth.^3^ With the growing interest surrounding aesthetic procedures for penile girth enhancement, many procedures have gained recognition, notably the use of a medical-grade silicone sleeve implant known as Penuma (rebranded Himplant, International Medical Devices, Beverly Hills, CA, USA), which has been demonstrated to improve retractile penis, penile shortening and narrowing from prosthesis surgery, and mild to moderate penile curvature.^4^^,^^5^ Some notable penile girth aesthetic procedures include the following: hyaluronic acid (HLA) injections,^2^ autologous fat injections,^6^ and implantation of a flap from the saphenous vein that expands the albuginea.^7^^,^^8^

The use of fillers began in 2003 when the FDA approved HLA products.^9^ For HLA injections, the procedure can be performed with local anesthesia. Ahn et al. describe that during their procedure, entry sites are typically located at the penile base at the 2 o’clock position and the distal penile shaft at the 10 o’clock position. Then, the filler is also injected between the Buck’s and Dartos fascia using a combination of fanning and back-and-forth techniques. Typically, ~15-22 ml is injected using this technique. During this study, there was a mean increase in flaccid penile girth of 22.74 ± 12.60 mm.^9^ In their review, Roth et al. concluded that while the literature on hyaluronic acid for penile aesthetics shows promising results, the quality of the studies varies.^10^

For autologous fat injections, Kang et al. conducted a study where they selected men whose proximal one-third and distal one-third of their penis had a mean thickness of 7.4 cm or less. These patients underwent fat suction with a liposuction device and then had fat injected into the superficial, middle, and deep layers of the Colles’ fascia. The injected fat volume ranged from 25-49 ml. After the procedures, the proximal third of the average patient’s flaccid penis was 9.39 ± 0.82 cm, and the average distal third was 9.34 ± 0.86 cm.^6^

Suspensory ligament utilization was documented by Li et al. in a study on dogs, where they used pericardium and blood vessel walls obtained from pigs.^8^ Incisions were made on the lateral sides of the penile bulb on the anesthetized dogs. Underneath the mucosa, blunt separation of the foreskin was performed, and this created a loop tunnel ~2 cm wide. The materials gathered from the pigs were implanted under the mucosa and subsequently sutured closed. Before surgery, the average flaccid penile girth was 7.30 ± 0.2 cm. One month post-operation, the average flaccid girth was 8.63 ± 0.46 cm.^8^ There are several non-invasive options available, such as vacuum devices and traction devices, as well as minimally invasive procedures like silicone and soft tissue fillers. More invasive options include grafts and flaps. Additionally, a mini-abdominoplasty or liposuction of the suprapubic fat pad can visually lengthen the penis. While non-invasive techniques are the least effective for increasing penile girth or length, minimally invasive and invasive options tend to offer better results, though they come with a higher risk of complications.^11^

In 2018, Dr. Elist reported the first use of Penuma, a silicone device that can be surgically inserted subcutaneously into the penile shaft to improve penile appearance.^5^ Registered with the FDA, the implant is customizable by the surgeon to aid in reconstruction and is available in three sizes with varying wall thicknesses, produced by International Medical Devices.^12^

Given the numerous options available, which procedures are patients mostly interested in and is there any regional trends in patient preferences?

## Materials and methods

A Google Trend (Google Inc., Mountain View, CA, USA) keyword search was conducted with the window of time being from June 18, 2018, to June 11, 2023. Search terms regarding penile girth enhancement procedures were used as keywords, with the following terms yielding the most results: “Penuma,” “penile implant,” “penile girth,” “HA injection,” and “penis injection.” To assess changes in search interest over time, trend lines were generated. Given its established reputation as a widely recognized penile enhancement procedure, Penuma’s search trends were used as a reference for comparison. The popularity of a search term was normalized to the number of searches for Penuma during the selected time interval. Search trends were graphically represented using trend lines. Because the data is normalized, it is easier to compare the relative popularity of searches. The numbers are scaled from 1-100 a.u. based on the topic’s proportion of all searches on all topics, with 100 a.u. representing the highest point of popularity within a topic. The initial search illustrated regional differences in penile girth aesthetic procedure preference that were also analyzed, and the regions were compared based on gross domestic product (GDP). Google Trends provides a random sample each time a search is conducted. It also does not send alerts whenever data has been updated. Because of this, the regional data may vary, but not drastically. To demonstrate this, the data for a larger window dating from June 18, 2018, to November 11, 2023, was included. The GDP of high-interest regions may explain regional differences, so the GDP for the metropolitan areas associated with high-interest regions was also included. This study does not require ethics approval or consent to participate.

## Results

**Figure 1:**
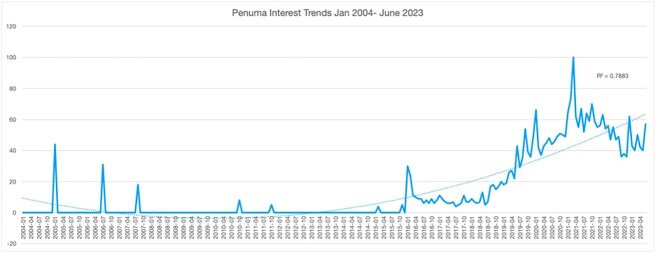
Penuma interest trends January 2004 to June 2023.

**Figure 2:**
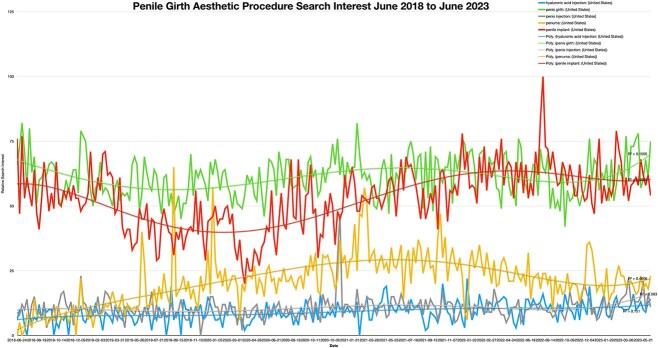
Penis girth aesthetic procedure search interest June 2018 to June 2023.

**Figure 3:**
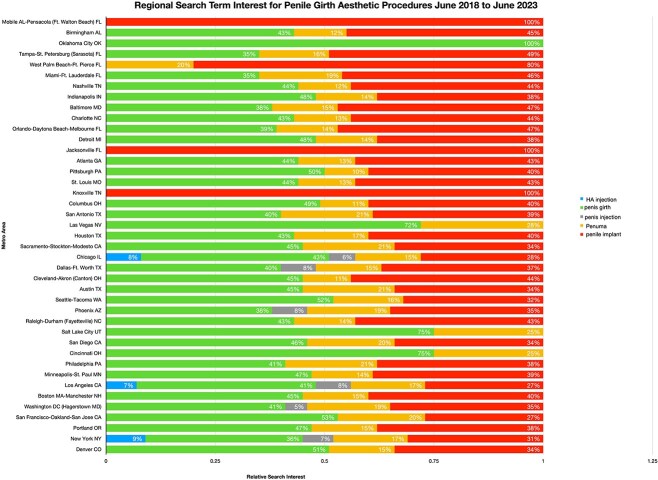
Regional search term interest for penile girth aesthetic procedures June 2018 to June 2023.

Data analysis showed that Penuma had some search interest when it first came to market in 2004 (Figure 1). Since then, interest has waned until March of 2021 (R^2 > 0.7). When looking at Penuma and comparing it with other terms that were searched between June 2018 and 2023, it was found that interest for general searches of “penile implant” and “penile girth” was significantly higher. However, search interest for Penuma was higher than that of HLA and penis injection terms, that had the least interest over this timespan (Figure 2). In detail, the relative search interest for the terms “penile girth” and “penile implant” consistently had a rolling average above 50 a.u. throughout the period. Additionally, “penile implant” reached the highest search interest at a single point, peaking at 100 a.u. on 7/22. However, the average search interest for “penile girth” was slightly higher than that of “penile implant.” Initially, “HA injection” and “penis injection” had relatively higher search interest than “Penuma”, but “Penuma” quickly surpassed them. Both “HA injection” and “ penis injection” maintained an overall average below 10 a.u., with values normalized based on Penuma’s search interest data (Figure 2). Figure 3 illustrates regional search interest for the penile girth aesthetic search terms. Penile implant interest was prevalent in Mobile, AL, Pensacola, FL, Jacksonville, FL, and Knoxville, TN. Penis girth was highly searched in Oklahoma City, OK, Salt Lake City, UT, and Cincinnati, OH. Penuma’s interest was highest in Las Vegas, NV, Salt Lake City, UT, and Cincinnati, OH. Searches for “penis injection” were high in TX, Phoenix, AZ, and Los Angeles, CA. The gross domestic product (GDP) for the corresponding metropolitan areas can be found in Table 1.

**Table 1:** Regional Areas and Associated GDP.

Metropolitan Statistical Area	All Industry total GDP in Thousands of Current Dollars (2022)
Mobile, AL	27 360 640
Pensacola, FL	27 079 985
Oklahoma City, OK	94 741 711
Jacksonville, FL	117 162 018
Knoxville, TN	58 892 855
Las Vegas, Nevada	160 727 901
Phoenix, AZ	362 086 516
Salt Lake City, UT	135 409 299
Dallas Fort-Worth, TX	688 928 266
Cincinnati, OH	186 141 091
Los Angeles, CA	1 227 469 203

U.S. Bureau of Economic Analysis, “CAGDP2 Gross domestic product (GDP) by county and metropolitan area 1” (accessed Tuesday, April 9, 2024).

Supplementary Figures 1 and 2 show search interest and regional interest from June 2018 to November 2023. This was done to assess how reliable the initial collected data was. Google Trends data changes as more information becomes available, so extending the time parameters would allow for any additions and/or changes in the data to be observed. Regional data from June 2018 to November 2023 was consistent with the regional data from June 2018 to June 2023.

## Discussion

To our knowledge, this is the first study to use internet search intent to measure interest in penile aesthetic procedures in the United States. This Google Trends study provides insights into consumer interest and regional variations in online searches for penile aesthetic procedures, with a specific focus on the Penuma implant. The analysis revealed notable trends and disparities, shedding light on public interest and its evolution over time.

The study revealed that search interest in Penuma initially surged in 2004 but declined until a resurgence in March 2021. Broader terms like “penile implant” and “penile girth” consistently attracted higher interest between June 2018 and June 2023, while “hyaluronic acid” and “penis injection” saw minimal activity. The regional analysis revealed distinct patterns, with each search term demonstrating peak interest in different metropolitan areas across the United States.

While this study demonstrates Penuma’s fluctuating popularity, prior research on penile aesthetic procedures has largely focused on clinical outcomes and patient satisfaction rather than public interest.^13^ Studies on penile implants and aesthetic enhancements typically highlight rising demand due to increased public awareness, suggesting that consumer interest often correlates with marketing efforts, availability, and regional socio-economic factors.^14^ However, the limited interest in newer technologies like HLA and penis injections contrasts with previous findings that emphasize the growing appeal of minimally invasive procedures in other aesthetic domains.^15^

The observed trends suggest that consumer interest in penile aesthetic procedures is influenced by both procedural novelty and geographical factors. The resurgence of interest in Penuma in March 2021 could be attributed to targeted marketing, media coverage, or advances in procedural safety and effectiveness.^16^^,^^17^ The regional disparities may reflect differences in socio-economic status, cultural norms, or accessibility to specialized providers.^18^ The correlation between metropolitan areas with high gross domestic product (GDP) and search interest underscores the role of economic factors in driving demand for elective procedures.^19^ The disparities could be driven by many factors that may influence consumer decision-making. One factor could be the possible effect of urologist density in the United States. An analysis of the American Urological Association Census compared urologists in rural areas to their urban counterparts and found that rural urologists are older and provide general urological care.^20^ This means that some regions of the country may have limited access to penile aesthetic procedures compared to regions with a higher density of urologists. This demonstrates a need to examine not only what procedures patients are familiar with but also what procedures or products are available in a specific region. Another factor driving consumer interest could be the GDP of the metropolitan area.^21^ Dallas-Fort Worth, TX, and Los Angeles, CA, have the two highest GDPs, and had high search interest in the term “penis injection.” It could be hypothesized that injections are more accessible in these areas compared to Mobile, AL, and Pensacola, FL, which had a higher preference for the general term “penis girth.” Using general terms rather than searching for the product by name may indicate that consumers want to know what options are available rather than having a specific procedure in mind. Areas with more general searches may receive less advertising than those with more targeted search terms.^22^ This is important because it may highlight the procedures that consumers are most familiar with. This data can be used by urologists to frame conversations they may have with patients interested in penile girth aesthetic procedures.

The trends are influenced by several additional factors. Patient motivations, particularly cosmetic desires and expectations, play a crucial role.^23^ The experience and reputation of doctors also have a significant impact on decision-making.^24^ Cost is another important consideration, as more affordable options may attract more patients, even if they carry higher risks or less favorable outcomes.^25^ Patient testimonials can boost visibility and drive demand for certain treatments. Additionally, patient satisfaction, shaped by the success or complications of the procedure, strongly influences the popularity of a given treatment.^26^ It’s also important to consider the impact of statements from organizations like the Society of Sexual Medicine in North America (SMSNA) and how these may influence both patient and physician choices, as well as search trends. The SMSNA strongly opposes the use of permanent materials like paraffin and silicone for penile fillers. The society encourages physicians to adhere to IRB-approved research protocols when using other injectables, such as HA and polyactic acid. Additionally, the SMSNA advises against the use of flaps and grafts for penile augmentation until more outcome data becomes available.^27^

The interest surrounding penile aesthetic procedures may not always be positive. With lawsuits concerning patient injury and/or dissatisfaction in regards to penile aesthetic procedures on the rise, this “bad press” may also influence peaks in patient interest when they are Googling penile aesthetic procedures. For example, in 2021, there was a class action lawsuit launched against the developers and marketers of Penuma. The complaint was that Penuma was falsely advertised as being a safe procedure in healthy men when it is FDA-approved for “cosmetic correction of soft tissue deformities”.^28^ A decision was never made regarding this class action because the plaintiff voluntarily dismissed the case. The reason why this was done is not specified, but court documents are available. Even though a decision was not made regarding this case, it is one of many negative results that can be found by merely typing “Penuma.” Advertisements can determine which results are seen first by consumers, but it is not possible to remove negative reviews or results if they are true occurrences.

## Strengths and limitations

A key strength of this study lies in its use of Google Trends data, which provides a real-time, population-level perspective on consumer interest. Additionally, the consistent findings across different timeframes validate the reliability of the data. The integration of GDP data provides a socio-economic context, linking search interest to regional wealth and economic activity.

However, there are several limitations to consider. Google Trends data reflects search interest but does not account for actual procedural uptake or consumer intent. Geographic interest could be skewed by variations in internet usage, population density, or local awareness campaigns. Additionally, the lack of demographic data, such as age or gender, limits deeper insights into the target population. Another limitation is that there is an infinite combination of search terms that a consumer may use when searching for penile girth aesthetic procedures. Terms such as HLA injections may have many implications because it is used in various specialties for many procedures; changes in the search trends for these terms may not reflect an actual interest in penile aesthetics.

## Future research directions

Future studies should aim to correlate search interest with procedural outcomes, patient satisfaction, and provider availability to better understand consumer behavior. Exploring the role of social media, advertising, and cultural perceptions in shaping interest in penile aesthetic procedures could provide additional context. Moreover, longitudinal studies tracking the impact of emerging techniques, such as HLA and other minimally invasive options, could offer valuable insights into shifting trends in the field.

## Conclusion

This study examines the trends in online search interest in Penuma compared to other terms related to penile enhancement. Although general search terms like “penile implant” and “penile girth” attracted more attention than Penuma, noticeable regional differences in search behavior exist. The results underscore the necessity for additional research to comprehend the factors influencing these disparities and their implications for consumer choices in various regions. These findings can help healthcare providers and policymakers design educational programs and allocate resources effectively to address the diverse needs and preferences of individuals interested in penile enhancement procedures.

## Supplementary Material

supplementary_1_qfaf013

supplementary_2_qfaf013

Supplementary_Figures_qfaf013

## Data Availability

Not applicable.
