# Supplementary figures and images for: Unveiling consumer interest and regional disparities: comparative analysis of online search trends for penile aesthetic procedures
Source: Sex Med. 2025 Mar 8;13(1):qfaf013. doi: 10.1093/sexmed/qfaf013 (PMC11890111; doi:10.1093/sexmed/qfaf013)

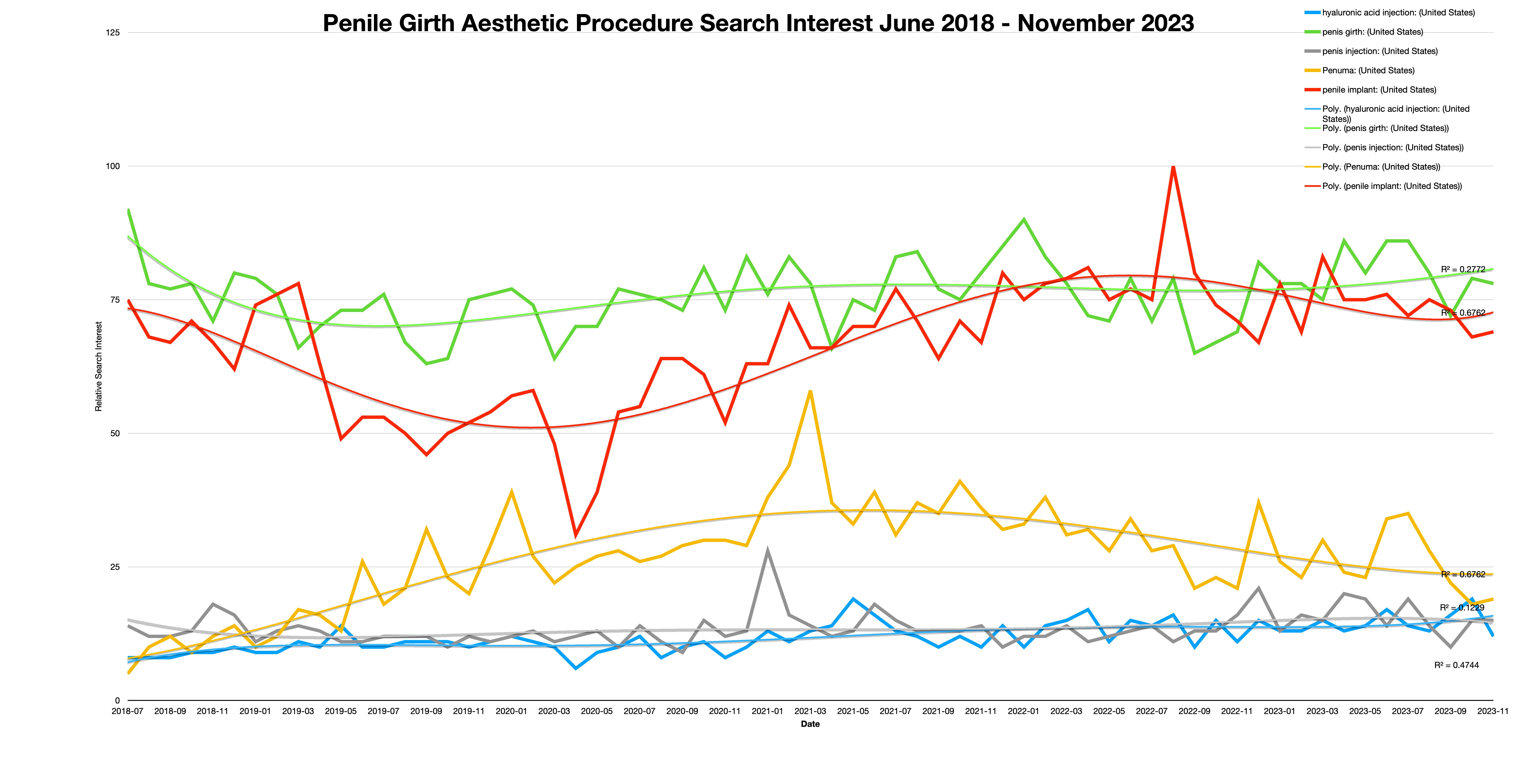

Supplement: supplementary_1_qfaf013 [file supplementary_1_qfaf013.jpeg]

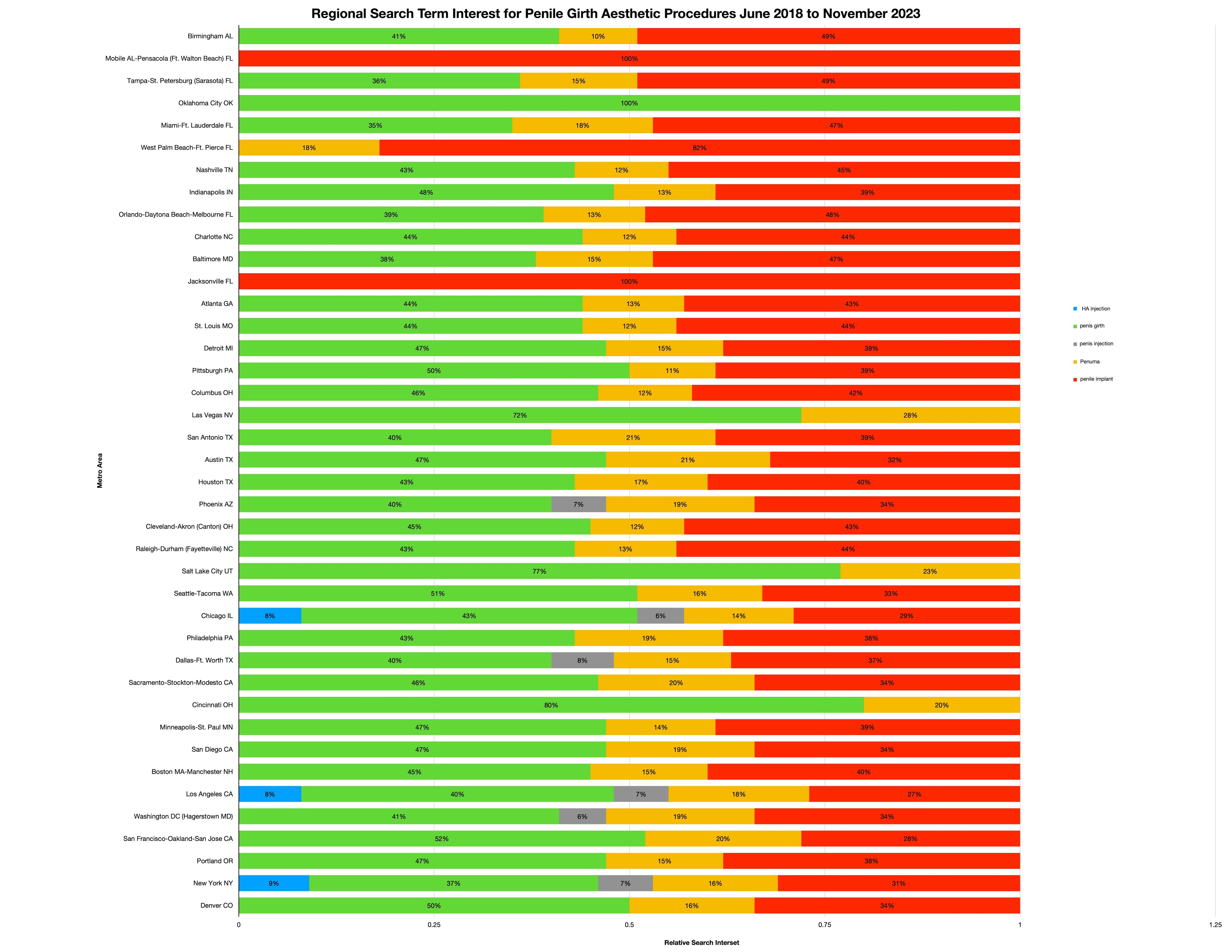

Supplement: supplementary_2_qfaf013 [file supplementary_2_qfaf013.jpeg]
