# Supplementary material for: Unveiling consumer interest and regional disparities: comparative analysis of online search trends for penile aesthetic procedures
Source: Sex Med. 2025 Mar 8;13(1):qfaf013. doi: 10.1093/sexmed/qfaf013 (PMC11890111; doi:10.1093/sexmed/qfaf013)
Supplement: Supplementary_Figures_qfaf013 [file supplementary_figures_qfaf013.docx]

Supplementary 1. Penile Girth Aesthetic Procedure Search Interest June 2018-November 2023

Supplementary 2. Regional Search Term Interest for Penile Girth Aesthetic Procedures June 2018 to November 2023
